# Dual-Use Strain Sensors for Acoustic Emission and Quasi-Static Bending Measurements

**DOI:** 10.3390/s24051637

**Published:** 2024-03-02

**Authors:** Jason Stiefvater, Yuhong Kang, Albrey de Clerck, Shuo Mao, Noah Jones, Josh Deem, Alfred Wicks, Hang Ruan, Wing Ng

**Affiliations:** 1 Department of Mechanical Engineering, Virginia Tech, Blacksburg, VA 24061, USA; 2 NanoSonic, Inc., 158 Wheatland Drive, Pembroke, VA 24136, USA

**Keywords:** acoustic emission, MEMS, strain sensor, source location, quasi-static plate bending

## Abstract

In this paper, a MEMS piezoresistive ultrathin silicon membrane-based strain sensor is presented. The sensor’s ability to capture an acoustic emission signal is demonstrated using a Hsu–Nielsen source, and shows comparable frequency content to a commercial piezoceramic ultrasonic transducer. To the authors’ knowledge, this makes the developed sensor the first known piezoresistive strain sensor which is capable of recording low-energy acoustic emissions. The improvements to the nondestructive evaluation and structural health monitoring arise from the sensor’s low minimum detectable strain and wide-frequency bandwidth, which are generated from the improved fabrication process that permits crystalline semiconductor membranes and advanced polymers to be co-processed, thus enabling a dual-use application of both acoustic emission and static strain sensing. The sensor’s ability to document quasi-static bending is also demonstrated and compared with an ultrasonic transducer, which provides no significant response. This dual-use application is proposed to effectively combine the uses of both strain and ultrasonic transducer sensor types within one sensor, making it a novel and useful method for nondestructive evaluations. The potential benefits include an enhanced sensitivity, a reduced sensor size, a lower cost, and a reduced instrumentation complexity.

## 1. Introduction

The sensing of the acoustic emissions (AE) released by damaging events, such as fatigue cracking, high-velocity impacts, or delamination, is commonly utilized to assess a structure’s integrity through nondestructive evaluations (NDEs) and structural health monitoring (SHM) [1]. The nondestructive nature of the testing lends itself to the assessment of permanent structures (e.g., bridges), for repetitive-use items which are subject to fatigue cracking (e.g., airplane wings), and for monitoring projectile impacts (e.g., spacecraft). Complex instrumentation is utilized to assess subsurface structural damage, which is unobservable to the naked eye, and to reduce the need for routine inspections. The introduction of such complex instrumentation has undoubtedly furthered the fields of NDEs and SHM, as well as having improved the safety of many crucial aforementioned industries. However, with continuous advancements in manufacturing and technology, advances in nondestructive testing and evaluation (NDTE) must keep pace. This calls for the improvement of testing instrumentation and data collection, with the goal of realizing a seamless connection within the requirements of modern technology [2].

The sensing of vibrations exists within the two following categories: active and passive measurements. The scope of this work focuses on measurements and analyses through the application of passive sensing, where sensors measure the excitation created by a controlled outside source [3,4]. A common tool utilized for both passive and active measurement within NDE is the piezoelectric ultrasonic transducer, which exists in a broad range of frequency bandwidths, typically operating between 200 kHz–10 MHz [5]. The sensor’s high-sensitivity and high-frequency response enables its use in capturing acoustic emission signals within complex structures. These low-energy, high-frequency stress waves are emitted by the propagation of surface level and sub-surface cracks and imperfections within a structure caused by the redistribution of strain energy [6,7,8]. The additional post-processing of these signals can reveal the approximate location of the AE source, therefore, AE sensing is particularly advantageous for locating sub-surface level cracking which is not captured during visual inspection. Many studies have been performed on the source location of AE events, and these contributions have aided in improving real-time structural health monitoring [7,8,9,10,11,12].

Piezoelectric sensors convert dynamic forces into electrical signals by means of the piezoelectric effect, and can accurately measure dynamic strain changes without latency [13]. However, this generated voltage quickly dissipates to the surroundings due to its easily neutralized surface charge [13,14]. This prevents the utilization of piezoelectric sensors in the sensing of static strain—an important measurement in aerospace and civil structures alike [15,16]. The use of piezoelectric ceramic materials, such as lead zirconate titanate (PZT), within transducers has enabled wider-frequency bandwidths and allowed broader applications [17,18,19,20]. Linsheng Huo et al. (2017) reported the utilization of a PZT transducer to closely match the signal of a strain sensor in a dynamic bending failure test of reinforced concrete. This demonstration highlighted the potential of PZT transducers in capturing lower frequency vibrations and, to some extent, measuring strains. This was achieved through a comparison between the signals obtained using a piezoresistive strain sensor [5]. However, the dynamic bending test was conducted with a quickly applied load (60 mm/s) and continued beyond the concrete’s elastic limit until fracture occurred. This testing method does not assess the performance of the PZT transducer for quasi-static bending strain measurement.

**Figure 1:**
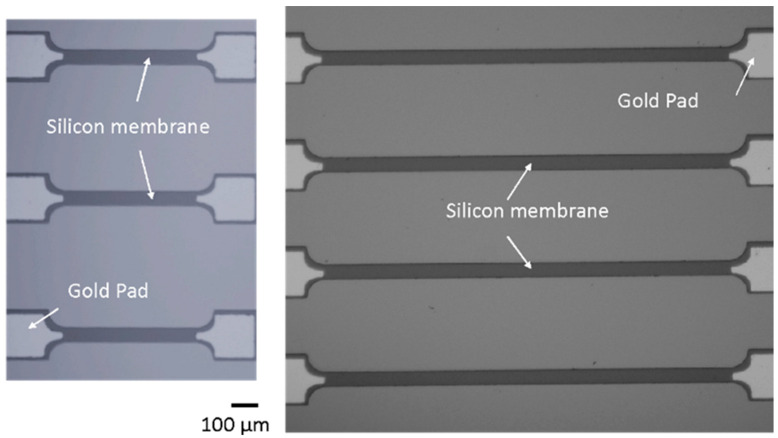
Microscopic images of the fabricated silicon membrane-based strain sensors of different sizes on SOI wafers, transferred to polyimide substrates for AE and strain measurements. The membrane thickness varies from a few hundred nanometers to a few microns.

**Figure 2:**
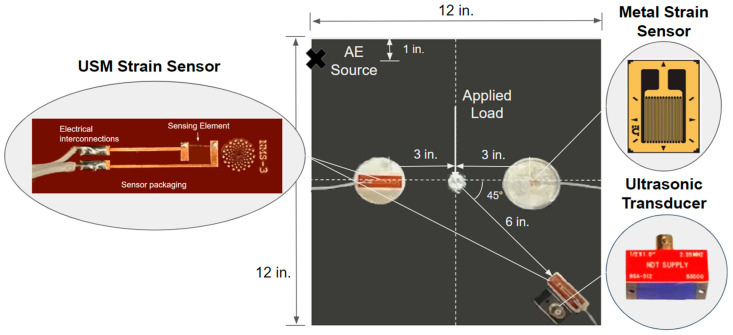
Schematic of the aluminum 6061-T6 testing plate with mounted piezoresistive ultrathin silicon membrane (USM)-based strain sensor and commercial sensors. Two USM strain sensors (sensing element: length/width/thickness: 500 μm/60 μm/4 μm) are mounted on the aluminum plate (2.2 mm thickness) with a Britek 2.25 MHz PZT ultrasonic transducer and a commercial Omega metal-based linear strain sensor (sensing element length: 11.8 mm). The bottom right USM strain sensor is utilized for acoustic emission experimentation, and the leftmost sensor for quasi-static bending experimentation.

In recent years, there have been studies utilizing piezoelectric films to measure acoustic emissions [21,22,23,24]; however, none have focused on low-energy acoustic emissions. In this study, the authors report the first experimental measurement of low-energy acoustic emissions using a piezoresistive MEMS strain sensor. Additionally, the sensor’s ability for the dual application of both quasi-static bending strain and high-frequency acoustic emission sensing is demonstrated. Shown in Figure 1 and Figure 2, the piezoresistive ultrathin silicon membrane (USM)-based strain sensor is fabricated on a silicon-on-insulator (SOI) wafer and transferred to a polyimide-based flexible substrate with electrical interconnections. The membrane thickness varies from a few hundred nanometers to a few microns, with the minimum lateral dimensions being at least two orders of magnitude greater than the thickness. The SOI-based silicon membranes are gaining acceptance in the semiconductor industry, as the benefits they provide have become key enablers in the scaling and performance enhancements of electric devices since the initial fabrication was introduced by John Rogers’ group [25]. At this time, the enhanced sensor fabrication process, which permits the co-processing of crystalline semiconductor membranes and advanced polymers to regulate the sensitivity and dynamic range of the sensor elements, is proprietary to NanoSonic Inc., Pembroke, VA, USA.

Typical metal and semiconductor piezoresistive strain sensors are historically reserved for static bending applications due to their large sensing element size and sensitivity, which restricts their frequency bandwidth and minimum detectable strain [26]. For this reason, these commercial strain sensors function well in measuring static bending strain, but fail to record low-energy, high-frequency AE. The presented miniaturized ultrathin silicon membrane-based strain sensor, fabricated by NanoSonic Inc., serves to bridge this gap in frequency bandwidth between ultrasonic transducers and strain sensors via the utilization of a piezoresistive micron-scale sensing element, which enables a wide-frequency bandwidth response and a low minimum detectable strain. The objective of the work herein is to demonstrate the NanoSonic strain sensor’s ability to reliably document low-energy, high-frequency AE signals, making it, to the knowledge of the authors, the first-known piezoresistive strain sensor technology capable of recording low-energy AE, as well as reliably document quasi-static bending strains. Through the dual monitoring of both bending strain measurements and AE events, the uses of multiple sensors can effectively be condensed into one strain sensor, thereby reducing costs, sensor size, and instrumentation complexities associated with NDE and SHM.

## 2. Materials and Methods

This section begins with a brief discussion of the necessary background information of the physics behind acoustic emissions in isotropic thin aluminum plates and quasi-static bending. Next, the experimental setup and necessary information on experiment replicability for the acoustic emission and bending tests are discussed. Additional information on the experimental setup can be found in the master’s thesis of Jason Stiefvater at Virginia Tech [27].

### 2.1. Methodology

#### 2.1.1. Acoustic Emissions in Isotropic Thin Aluminum Plates

Within plate-like elements (where the thickness is considerably less than the other two plate dimensions), Lamb waves are the dominant mode of AE propagation [9]. These waves occur as two basic modes as follows: symmetric (*S_o_*) and asymmetric (*a_o_*) (also referred to as extensional and flexural modes, respectively) [12]. Higher order modes can exist, but are typically low energy, particularly in thin plates (where AE wavelengths are much larger than plate thickness), and insignificant in AE applications [8]. An important attribute of the extensional (*S_o_*) wave is that it acts as a precursor to the flexural wave, and is essentially non-dispersive; however, it is typically a much lower amplitude and a higher frequency. The non-dispersive nature of the extensional wave makes it much easier for time-of-flight AE source location techniques to be applied, but its low-amplitude and high-frequency content makes it difficult to sense. The flexural (*a_o_*) wave, however, often has a much greater amplitude and is highly dispersive (meaning its frequency content separates with time, since the higher frequencies propagate at a higher velocity). Due to the inherent tradeoffs between the source location techniques of the extensional and flexural modes, an accurate recording of both AE modes is desired. These acoustic emission waves within thin plates have been extensively studied and characterized, and their respective frequency content has been documented using PZT ultrasonic transducers [28]. For these reasons, a thin aluminum plate was utilized to study the USM strain sensor’s performance in sensing AE. 

The pencil lead break (PLB), commonly referred to as a Hsu–Nielsen source which is based on the popularization of the method by Hsu and Nielsen, is a long-established standard as a reproducible artificial AE source within thin plates [29]. The PLB resembles real crack formation/AE phenomenon, and produces both extensional and flexural AE modes within thin plates [11]. This technique is used in the present experimentation in order to repeatably generate AE within a thin aluminum plate.

#### 2.1.2. Quasi-Static Plate Bending

In this experiment, a slowly increasing load is applied to the center of the plate to apply quasi-static bending. Within solid mechanics, the quasi-static bending of a plate implies that the loading occurs at a slow enough rate where the inertial effects and forces are negligible. At the macroscopic level, the stress versus strain curve under quasi-static loading now monotonically increases, meaning the relationship only increases with respect to one another [30]. Assuming small plate deflections where loading occurs only in the elastic region, and the applied load increases linearly with time, both the bending stress as a function of strain and the strain as a function of time increase linearly [31,32]. The resulting sensor outputs for this experiment, as the load linearly increases, should be linear, meaning that tension causes an increase, compression causes a decrease.

### 2.2. Experimental Setup

#### 2.2.1. Acoustic Emission Testing

**Figure 3:**
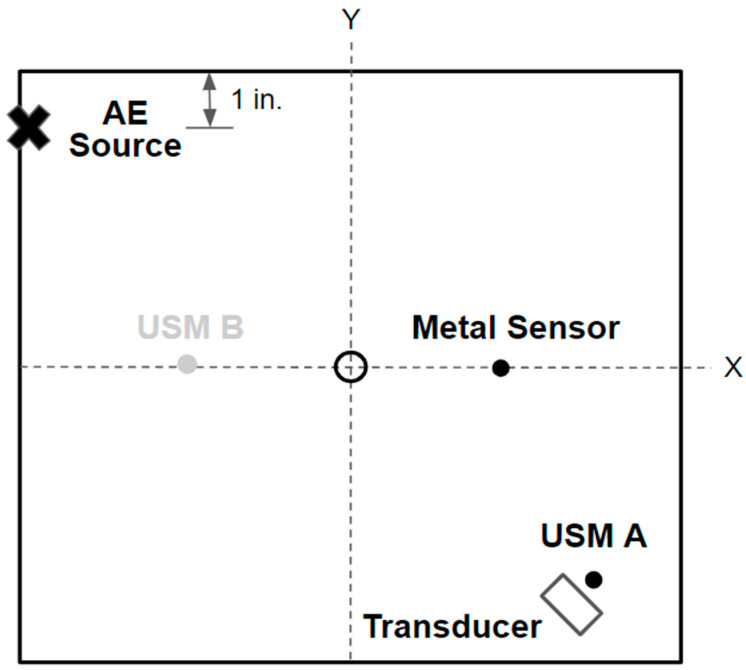
Schematic representation of the acoustic emission testing experimental setup. Testing was performed on some aluminum 6061-T6 12-inch square plate with 0.087-inch thickness.

The position of the acoustic emission source (PLB) relative to the sensors is shown in Figure 3. This position was chosen as it was farthest away from the previously mounted USM sensor; the location was offset an inch lower in the Y axis in order to avoid interference from the center plate hole. USM sensor “A” was also chosen for AE experimentation as its location allows for a farther AE source, which allows for the greater separation of the extensional and flexural AE modes [9]. The PZT ultrasonic transducer was placed next to the USM strain sensor “A” in such a way that both sensing elements would be subjected to the traveling acoustic emission stress waves at approximately the same time from a source approximately 14 inches away. 

**Figure 4:**
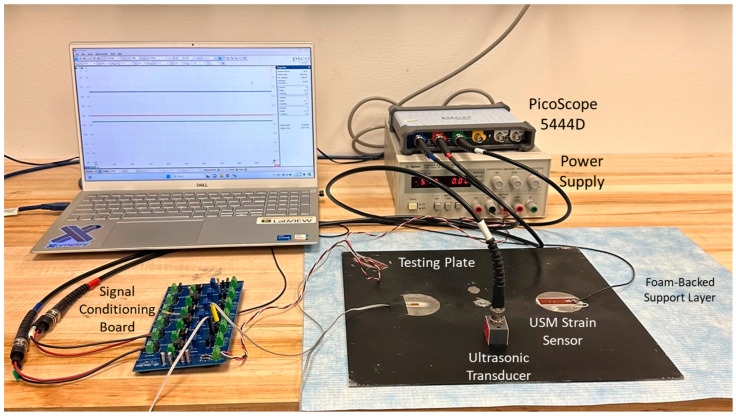
Acoustic emission experimental testing setup. Note that the ultrasonic transducer could be fixed to various locations depending on the desired setup.

Figure 4 shows the experimental setup utilized in the acoustic emission testing. The thin aluminum plate with the mounted strain and ultrasonic sensors is laid flat on a foam-backed support layer. The strain sensors (USM- and metal-based) signals went directly to the signal conditioning board, which was supplied with ±5.7 V of power by an external power supply. The board is based on a Wheatstone bridge configuration to convert the change in strain sensor resistance to an amplified output voltage. Strain sensor leads are taken as the input (USM- or metal-based), and the board utilizes a potentiometer to balance the bridge. An INA118 instrumentation amplifier is used with a ~1 kΩ gain resistor in order to set the amplification gain to approximately 51 for the output signal. The output of the signal conditioning board is sent to the PicoScope 5444D for data acquisition. The ultrasonic transducer output went directly to the PicoScope. We collected data for 50 ms at 17.86 MHz to ensure the entire AE signal and any subsequent reflections were captured, and that any aliasing effects were avoided. A rising slope trigger was placed on the transducer signal to begin the data acquisition. 

#### 2.2.2. Quasi-Static Plate Bending Testing

**Figure 5:**
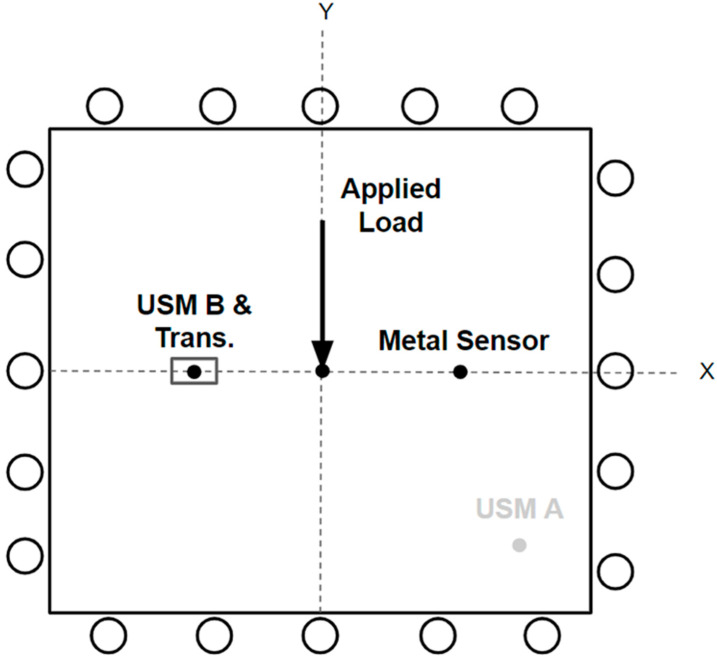
Schematic representation of the bending strain testing experimental setup. Loading was performed perpendicularly (along the Z-axis) to some aluminum 6061-T6 12-inch square plate’s center with a thickness of 0.087 inches.

**Figure 6:**
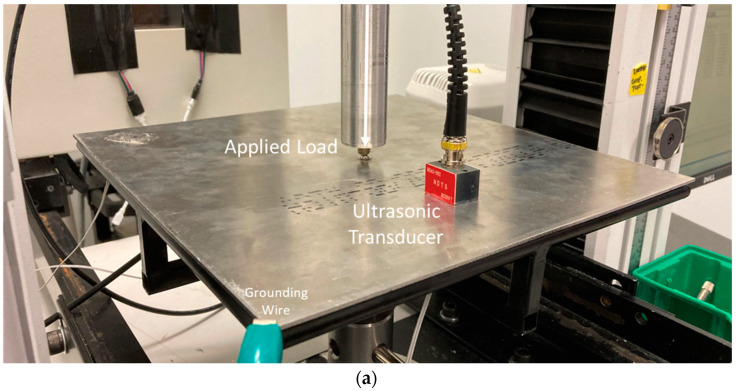

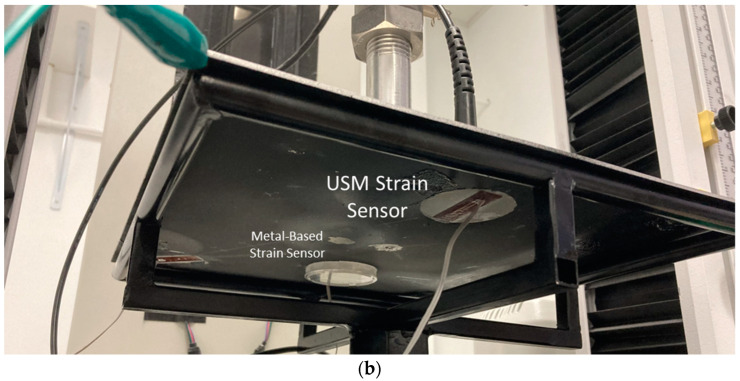
Quasi-static plate bending test experimental setup. Note that the plate was inverted to allow for all sensors to be subjected to approximately the same bending strain. (**a**) top isometric view, (**b**) bottom isometric view.

Figure 5 presents a schematic for the quasi-static plate bending experimental setup, where a simulated point load is applied perpendicularly to the plate’s center using an Instron 3369 machine. The plate rests on a custom-made static plate holder to provide a simply supported plate boundary condition. Since USM sensor “B” is closer to the center loading of the plate, it is subjected to a greater bending stress and, therefore, outputs a greater response to the loading. For this reason, USM sensor “B” was utilized for this test. To ensure both the USM strain sensor and the ultrasonic transducer are subject to the same loading, the plate was flipped upside-down, and the transducer was placed directly over top of the USM sensor, as illustrated in Figure 6. The difference in tension versus compression is accounted for in post-processing by multiplying the transducer data by a negative one.

The strain sensor signals (USM and metal) are routed to a NanoSonic Inc. signal conditioning box. The box uses a similar Wheatstone bridge configuration with potentiometers to output strain sensor signals, as well as an upgraded INA849 instrumentation amplifier. It houses its own internal power supply, and, because of this, the output voltage is added with additional noise. The NanoSonic team is working on another signal conditioning box with an internal power supply that has reduced noise and an improved signal/noise ratio. Output signals from this box are sent to a PicoScope 5444D for data acquisition. The data were sampled at 200 kHz for 5 s for the 1 mm/s and 2 mm/s tests, 500 kHz for 2 s for the 4 mm/s test, and 1 MHz for 1 s for the 8 mm/s test. A rising slope trigger was placed on the USM sensor signal to begin the data acquisition.

## 3. Results and Discussion

Once the experimental data were collected, MATLAB (Version 9.7 R2019b) was utilized for all data processing. MATLAB’s Signal Processing Toolbox was used for cross-correlation and power spectral density calculations. The analysis of the respective sensor responses to the acoustic emissions was split into signal similarities within the time and frequency domains. The results of the respective sensor performances to acoustic emissions and quasi-static bending are described in this section.

### 3.1. Sensor Response to Acoustic Emission

#### 3.1.1. Time Domain Analysis

**Figure 7:**
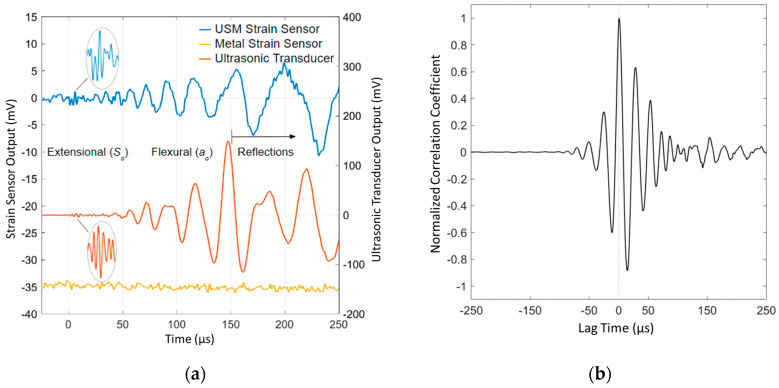
Time domain analysis of the USM strain sensor response to acoustic emissions compared to an ultrasonic transducer generated by a Hsu–Nielsen source. (**a**) The recorded AE signals plotted as a function of time. The USM strain sensor signal (blue trace) and the commercial metal-based strain sensor (yellow trace) are plotted on the same left *y*-axis scale; the commercial 2.25 MHz broadband ultrasonic transducer (orange trace), is plotted on the right *y*-axis scale. Measured AE signal characteristics (extensional and flexural modes) are labeled along with subsequent plate-end reflections, beginning at approximately 150 μs after the first strain arrival. The signals were digitally filtered with a Gaussian-weighted moving average filter. (**b**) Normalized cross-correlation between the filtered AE signals of the USM strain sensor and the ultrasonic transducer for the 100 μs preceding and 150 μs following the first strain arrival.

Notably, the USM strain sensor captures the AE generated by the Hsu–Nielsen source, as well as the ultrasonic transducer. Figure 7 shows the filtered recorded signal of both sensors. The USM strain sensor documents both the flexural (*a_o_*) and extensional mode (*S_o_*) from the AE source. The small-amplitude, high-frequency extensional mode is seen immediately following the zeroed time of strain arrival, which is succeeded by the larger amplitude flexural mode. The USM strain sensor’s apparent ability to record the extensional wave makes it a strong candidate to replace ultrasonic transducers in AE sensing, as it can be used for extensional wave-based time-of-flight AE source location. A more complex method of AE source location in thin plates, demonstrated by Gorman and Ziola, utilizes the dispersive flexural wave and cross-correlation, further validating the USM strain sensor’s potential in AE source location [9].

Between the times of the strain arrival and approximately 150 μs, the ultrasonic transducer and USM strain sensor capture nearly identical signals, barring noise in the USM strain sensor signal. This noise is presumably a result of electromagnetic interference (EMI) from the surrounding laboratory instrumentation and the sensor signal conditioning board. The NanoSonic Inc. team is already working to develop a more EMI-resistant sensor packaging and signal-conditioning unit to improve the sensor’s signal/noise ratio. The cross-correlation between the filtered USM strain sensor and ultrasonic transducer AE signals in Figure 7b further illustrates the strong similarity in documented signals. A maximum correlation is observed at approximately zero lag time, demonstrating that the strongest correlation between sensor signals occurs at the zeroed time of strain arrival. This verifies the time resolution and sensitivity of the USM strain sensor as it begins signal excitation at the same time of th strain arrival as the ultrasonic transducer, as both sensors were equidistant from the AE source. Following 150 μs, plate-end reflections begin influencing the recorded signals. Previously seen in Figure 2, the ultrasonic transducer is placed directly next to the USM strain sensor, which is mounted on the plate’s diagonal. Since the two sensors are in slightly different positions relative to the plate edges, they are subject to different plate-end reflections and, therefore, capture different signals after the 150 μs. The commercial metal-based strain sensor is unable to provide any significant response to the AE excitation, due to a limited frequency bandwidth as a result of its large sensing element size.

#### 3.1.2. Frequency Domain Analysis

**Figure 8:**
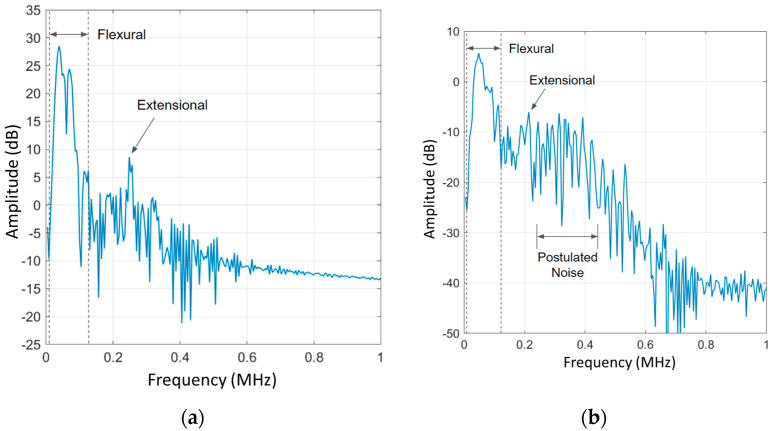
PSD of the filtered AE data from both sensor types, where 0 dB is 1 mW into 1 Ω. (**a**) Ultrasonic transducer PSD of recorded AE with labels denoting flexural vs. extensional mode frequency content. (**b**) USM strain sensor PSD of recorded AE. High-frequency content beyond the extensional mode frequency range is attributed to EMI in the sensor signal and noise in the signal conditioning board [27].

Additional analyses were performed to compare the frequency content of the USM strain sensor and the ultrasonic transducer’s respective AE signals, and to verify their similarities; the metal strain sensor is omitted from frequency analysis due to its non-responsiveness to AE. Figure 8 begins this comparison by plotting the power spectral density (PSD) of the filtered sensor signals. The transducer results are consistent with AE spectral results completed by Gorman (1991), with the spectral plot consisting of two distinct regions as follows: a higher-energy, lower-frequency flexural mode, and a lower-energy, higher-frequency extensional mode of vibration present within the AE signal [29]. These results are expected from the transducer as it has been previously shown to be a reliable tool in capturing flexural and extensional modes of vibration, thus making them a qualified benchmark for comparison in AE sensing. Notably, the USM strain sensor shows very similar frequency windows as the transducer. The USM sensor records a flexural mode window, spanning approximately 17.4–122 kHz, comparable to the transducer’s 17.4–131 kHz. The nearly identical flexural mode frequency content further validates the accuracy of the flexural mode AE signal captured by the USM strain sensor.

The two sensors also capture comparable extensional mode frequency content. The transducer captures a distinct extensional mode peak at 249 kHz, whereas the USM sensor records one at 214 kHz. Similar, but not identical, one potential cause for this discrepancy in extensional mode peak frequency is distortion in the USM strain sensor signal due to the previously mentioned high-frequency EMI and noise in the sensor’s signal conditioning unit. The noise can be seen in Figure 8b, directly following the extensional mode peak from approximately 248–458 kHz. A PSD of the signal noise preceding the AE signal arrival demonstrates the same frequency content of 248–458 kHz [27]. No peaks were observed at 214 kHz, which indicates that the 214 kHz peak is a deterministic signal amongst the high-frequency noise, and this peak is the result of the low-energy extensional AE mode. As previously stated, work is currently being conducted to reduce EMIs and improve the sensor’s signal conditioning unit to further increase the sensor’s signal/noise ratio. With these improvements, the sensor is expected to capture a refined extensional mode signal. Nevertheless, the frequency content of the extensional mode is not used within extensional mode AE source location techniques, and only the extensional mode time-of-flight between sensors is utilized due to its non-dispersive nature [10]. Therefore, the discrepancy in extensional mode frequency content does not hinder the USM strain sensor’s potential for extensional mode-based AE source location. Flexural mode source location techniques do, however, rely on an accurate sensing of the frequency dispersion within the plate [28]. As demonstrated by the nearly identical flexural mode frequency content between sensors, the USM strain sensor can also be potentially used in flexural mode AE source location. These findings illustrate the viability of the USM strain sensor’s ability to perform both extensional and flexural-based AE monitoring and source location, a crucial task within NDE and SHM, currently performed by ultrasonic transducers. The sensor’s wide-frequency bandwidth and low minimal detectable strain allow this to be the first piezoresistive-based strain sensor capable of low-energy AE sensing; additionally, these characteristics allow for the sensing of other important nondestructive structural health measurements, such as bending strain.

### 3.2. Sensor Response to Acoustic Emission

To have a true “dual application” sensor capable of both AE and bending strain measurements, it is necessary for the sensor to operate on a wide-frequency bandwidth. The USM strain sensor’s ability to document low-energy, high-frequency acoustic emissions was demonstrated in the previous section; however, to evaluate the sensors’ responses to bending strains, both sensors were subjected to quasi-static loading at various loading rates. This, in turn, evaluates their potential for wide bandwidth, dual sensing applications. 

**Figure 9:**
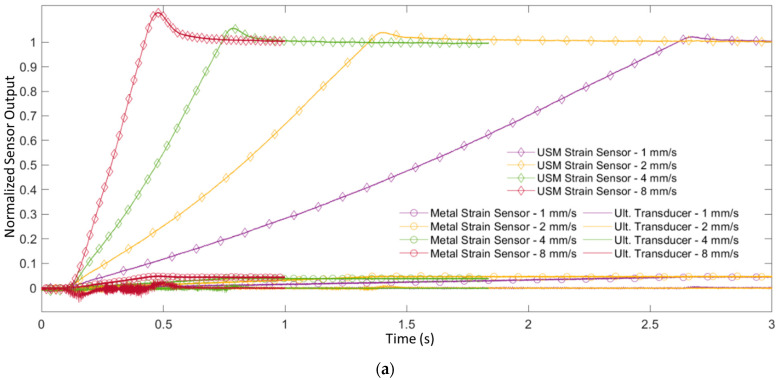

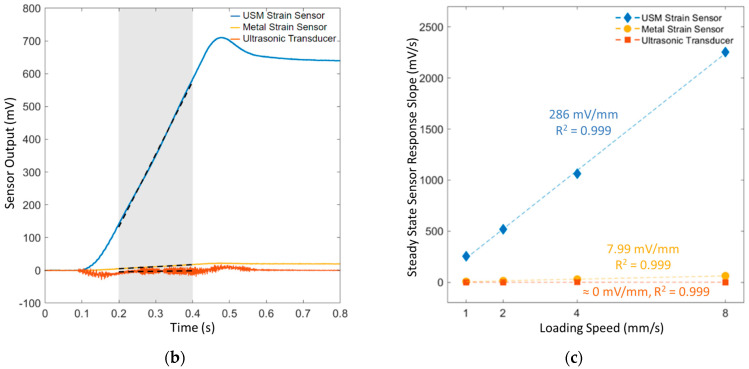
Bending strain performance of the USM strain sensor, metal-based strain sensor, and ultrasonic transducer when subject to quasi-static bending. (**a**) 500 Hz lowpass filtered responses of the sensors subject to plate bending across various constant loading rates up to a maximum load of 250 N. The *y*-axis is normalized to the USM strain sensor steady state output at a load of 250 N. Diamond and circular plot markers are utilized on response traces to differentiate between the USM and metal strain sensor responses, respectively; the ultrasonic transducer traces are solid lines with no markers. (**b**) The time history of the three sensor responses to the 8 mm/s applied load, highlighting the region of quasi-static loading from 0.2–0.4 ms where no acceleration of the loaded plate is present. A linear line of best fit for all respective sensor traces are plotted in this region. (**c**) The calculated slope of the sensor responses in the quasi-static loading region, plotted as a function of the applied loading rate for the USM strain sensor (blue), metal strain sensor (yellow), and the ultrasonic transducer (orange), along with a best fit line and coefficient of determination calculated for all data sets.

The respective sensor responses to this loading are shown in Figure 9a. The USM strain sensor outputs a steady linear response to the constant deformation of the applied load across the four loading rates. A clear differentiation in sensor response slope is noticed across the various loading rates, due to the sensor’s sensitivity to bending. The commercial metal-based strain sensor documents a similar linear response to the loading, but at a lesser magnitude due to the lower gauge factor. Additionally, the overshot of the applied load is captured for each loading rate test. This overshoot, which is seen to be greater in the faster speed tests, is caused by an overshoot in the prescribed maximum load by the load frame machine; this is confirmed by the respective loading curves [27].

The ultrasonic transducer, however, only provides inertial responses. The limited response is due to the piezoelectric sensors’ ability to measure rapid strain changes and inability to retain surface charge [13,14]. By the nature of this testing, some inertial effects will be present at the beginning and ending of loading, due to the acceleration and deceleration of the plate. This implies that the loading test is not entirely quasi-static, particularly in the faster applied loads. The transducer captures the respective loading dynamics at the beginning and ending of loading, but provides no significant response during steady-state, quasi-static bending. Figure 9b demonstrates this comparison between the sensors’ responses by highlighting the steady-state loading region from 0.2–0.4 ms for the 8 mm/s bending test (shaded area in Figure 9b), where bending is thought to be truly quasi-static. The calculated linear best-fit lines across this steady-state loading region show the USM strain sensor’s strong response to quasi-static bending and the PZT ultrasonic transducer’s flat response.

To further analyze the respective sensors’ performance with bending strains, Figure 9c plots the respective sensor response slope during this region of quasi-static loading as a function of the applied loading rate. The resulting data points for the USM strain sensor are used with a simple linear regression model to create a linear best fit line with a slope of 286 mV/mm, denoting a great change in the sensor response as a function of plate displacement. The regression model is met with a strong linear correlation with a coefficient of determination (R^2^) of 0.999, demonstrating the USM strain sensor’s sensitivity to the bending strain and in differentiating loading rates. The metal-based strain sensor also demonstrates the ability to differentiate the loading rates a high degree of accuracy, achieving an R^2^ of 0.999. However, it exhibits sensitivity levels over an order of magnitude lower compared to other sensors, with a linear best fit slope of 7.99 mV/mm. The ultrasonic transducer, however, provides a near-zero linear best fit slope with an R^2^ of 0.311, denoting little-to-no correlation in sensor responses and applied loading rates. This testing further demonstrates the transducer’s inability for quasi-static bending strain sensing (and therefore, its inability in the dual application sensing of both high-frequency and quasi-static loading cases), and further highlights the USM sensor’s potential for dual-application sensing.

## 4. Conclusions and Future Work

The presented USM strain sensor aims to progress the fields of NDE and SHM by improving the way strains are recorded and analyzed when monitoring the health of a structure. Typically, a distributed network of permanently attached sensors, often including ultrasonic transducers and strain sensors, are employed to accomplish this [33]. The presented USM strain sensor has been demonstrated to effectively combine the functionalities of both strain sensors and ultrasonic transducers by providing a wide-frequency bandwidth response with a low minimum detectable strain. This includes an improved response to bending strains in the form of quasi-static bending, typically characteristic of a piezoresistive strain sensor, and a comparable response in high-frequency AE sensing, typically monitored by ultrasonic transducers. This demonstration has shown the USM strain sensor to be the first known piezoresistive strain sensor which is capable of sensing an accurate low-energy AE signal, including its low-energy, high-frequency extensional mode. Measuring dynamic surface strains is impacted by the physical size of the sensing element—the so-called spatial filtering effects. For AE applications, the frequency is high, and the wavelength is short, therefore, the small dimension of the USM sensors makes them appealing. The dual monitoring of quasi-static loading and high-frequency acoustic emissions enables the sensor for use as a permanent fixture to a structure in order to simultaneously provide in situ bending strain measurements and monitor AE events, such as fatigue cracking, high velocity impacts, and delamination, potentially source-locating such events. These qualities all work towards the ability to provide real-time evaluations of the health of the structure. The combination of the traditional strain sensor and ultrasonic transducer sensing capabilities within one singular miniaturized sensor can provide significant and novel improvements to the fields of NDE and SHM by not only reducing cost and instrumentation complexities, but by further advancing these fields through the application of modern MEMS technology. 

Cross-sensitivities, such as temperature, affect the sensor elements due to environmental parameters other than strain and AE events, which were initially observed. The team has incorporated a temperature-sensing layer inside the material, thus allowing the measurement of temperature independently, and allowing calibration. The feasibility of using a thermo-sensing layer inside the materials as temperature sensors allows for the direct compensation of the material temperature for the static and dynamic strain measurements.

The natural progression of the future of this work is to perform the AE source location of PLBs, as well as the source location of impact events. These areas are currently being explored, and the preliminary results of both source location techniques are generally positive, as seen in the Appendix. Another area of interest is fatigue monitoring. Gorman (1991) has previously demonstrated the ultrasonic transducer’s capability in differentiating fatigue cracking events from noise events in a test specimen undergoing realistic flight fatigue loading [7]. If the USM strain sensor were to be shown to be capable of making these same measurements, it would further strengthen its claim in being a novel NDE/SHM sensor which is capable of a wide variety of in situ measurements that can assess the health of a given structure, all the while condensing the need for multiple sensor types, thus simplifying and improving sensor performance. 

## Figures and Tables

**Figure A1:**
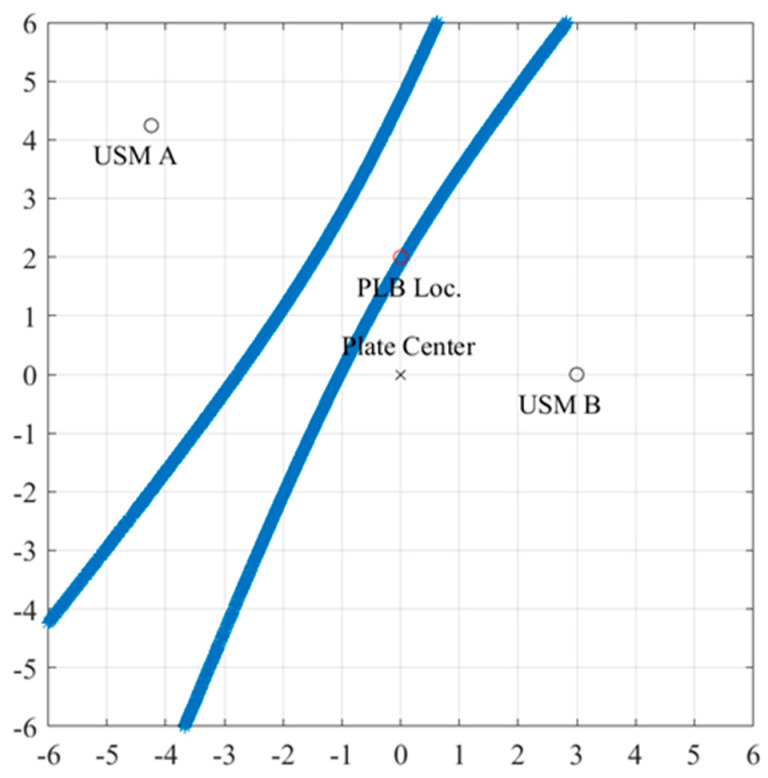
The “bilateration” of an AE source (PLB) on a 12 × 12 × 0.087-inch aluminum 6061-T6 plate using an extensional mode time-of-flight source location technique. The potential AE source locations are reduced to two hyperbolae. The resulting error is less than an eighth of an inch.

**Figure A2:**
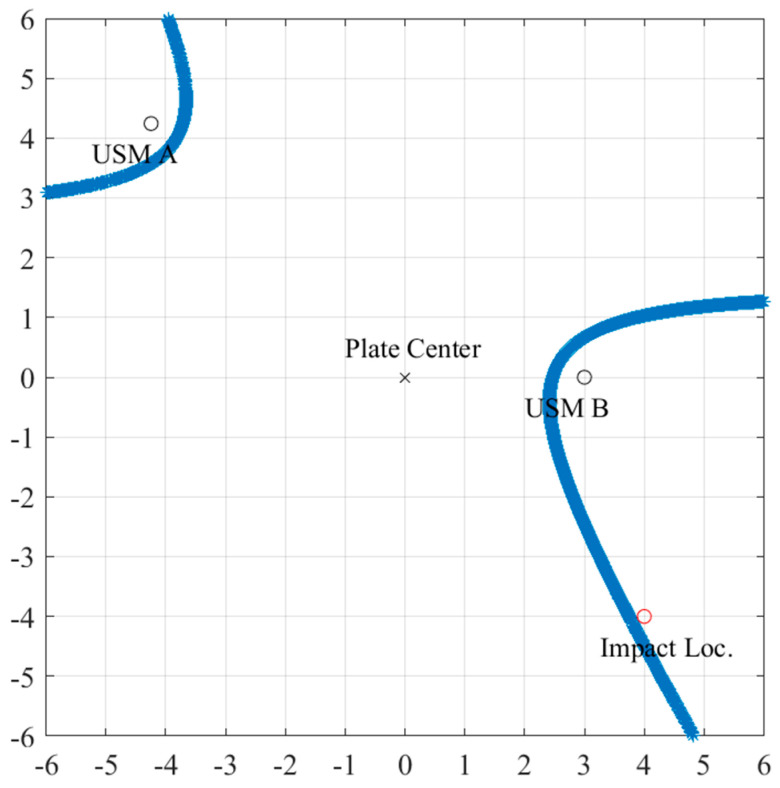
The “bilateration” of a high velocity impact (2.16 g ball bearing) on a 12 × 12 × 0.087-inch aluminum 6061-T6 plate using a flexural mode cross-correlation-based AE source location technique. The potential impact locations are reduced to two hyperbolae. The resulting error is about a quarter inch.

## Data Availability

The data presented in this study is available on request from the corresponding authors.

## Appendix A. AE Source Location Preliminary Results

This section presents the promising preliminary work performed on the source location of acoustic emission events using the USM strain sensors. Due to limitations in sensor quantities (due to their manufacturing complexities and uses in other programs), only two USM strain sensors have been mounted to the testing plate thus far. Tobias (1976) approached the problem of AE source location as three intersecting circles, each of which are centered at the receiving sensor, and intersect at the AE source location [10]. Since only two sensors are mounted on the plate (and trilateration techniques require three sensors), the derivation of the AE source location equation can be reworked to show that, for isotropic materials, the AE source location can be shown to lie on a hyperbola, with the two sensors as foci [9]. The resulting equations for the x and y coordinates of the hyperbola of potential AE source locations are shown in Equations (A1) and (A2):

(A1) $$x\left(\theta \right)=\left[\frac{x_{1}^{2}+y_{1}^{2}-\delta_{1}^{2}}{2\left(x_{1}\cos\theta +y_{1}\cos\theta +\delta_{1}\right)}\right]\cos\theta$$

(A2) $$y\left(\theta \right)=\left[\frac{x_{1}^{2}+y_{1}^{2}-\delta_{1}^{2}}{2\left(x_{1}\cos\theta +y_{1}\cos\theta +\delta_{1}\right)}\right]\sin\theta$$

where *x*_1_ and *y*_1_ are the coordinates of sensor 2 relative to sensor 1. *δ*_1_ = Δ*t × V,* where Δ*t* is the time-of-flight difference of the strain extensional mode arrival between sensors, and *V* is the velocity of the extensional mode in aluminum. The resulting *x* and *y* coordinates are calculated for all values, spanning zero to 2π radians. The preliminary results of the AE source location of a PLB using this technique with the USM strain sensors are shown in Figure A1. The hyperbola of potential AE sources is off by less than an eighth of an inch; these results are comparable with the calculations performed using ultrasonic transducers as a baseline test.

This technique, however, operates on the time-of-flight differences between sensors of the arrival of the extensional AE mode due to its non-dispersive nature. The extensional mode is typically much lower in amplitude than the subsequent flexural mode, and is often damped out or not seen in noisy systems. Gorman and Ziola (1991) published a method of AE source location in isotropic thin plates using cross-correlation, which can be used to perform AE source location on significantly larger flexural modes [9]. This technique involves cross-correlating the flexural mode AE signal with a cosine function modulated by a Gaussian pulse. The modulated cosine function follows Equation (A3) as follows:

(A3) $$x\left(t\right)=e^{-\left[{\left(t-t_{1}\right)}^{2}/\sigma^{2}\right]}\cos\left(\omega_{1}t\right)$$

where ω_1_ = 6.283 × 105 rad/s (f_1_ = 100 kHz), t_1_ = 100 μs, and σ = 40 μs. These values were chosen to best match the initial flexural mode which arrives at the sensor before reflections interfere with the signal. The sensor signal and Equation (A3) are cross-correlated, and the subsequent output function essentially isolates the time at which the chosen frequency (in this case 100 kHz) is present. The time-of-flight differences between the sensors can then be used in Equations (A1) and (A2). This process was performed for a high-velocity impact of a 2.16 g ball bearing on the plate’s surface, and the resulting “hyperbolic bilateration” is shown in Figure A2. The resulting preliminary results are off by about a quarter inch, which is very promising for the potential success of the source location of an AE event with the addition of a third USM strain sensor.
